# Endoscopic Ultrasound-Guided Sampling for Personalized Pancreatic Cancer Treatment

**DOI:** 10.3390/diagnostics11030469

**Published:** 2021-03-08

**Authors:** Eisuke Iwasaki, Seiichiro Fukuhara, Masayasu Horibe, Shintaro Kawasaki, Takashi Seino, Yoichi Takimoto, Hiroki Tamagawa, Yujiro Machida, Atsuto Kayashima, Marin Noda, Hideyuki Hayashi, Takanori Kanai

**Affiliations:** 1 Division of Gastroenterology and Hepatology, Department of Internal Medicine, Keio University School of Medicine 35, Shinanomachi, Shinjuku-ku, Tokyo 160-8582, Japan; masayasu.horibe@gmail.com (M.H.); s.kawasaki@keio.jp (S.K.); seinosann@gmail.com (T.S.); takimoto.yo1@gmail.com (Y.T.); h.tamagawa@keio.jp (H.T.); machiyumachiyu@keio.jp (Y.M.); shimauma@keio.jp (A.K.); jesuis_intense@yahoo.co.jp (M.N.); takagast@keio.jp (T.K.); 2 Center for Diagnostic and Therapeutic Endoscopy, Keio University School of Medicine 35, Shinanomachi, Shinjuku-ku, Tokyo 160-8582, Japan; fukuharas@keio.jp; 3 Keio Cancer Center, Genomic Units, Keio University School of Medicine 35, Shinanomachi, Shinjuku-ku, Tokyo 160-8582, Japan; rock-hayashi-pop@keio.jp

**Keywords:** EUS-guided sampling, pancreatic cancer, patient-derived organoid, precision medicine

## Abstract

Pancreatic cancer is the most lethal solid malignancy, and the number of patients with pancreatic cancer is increasing. Systemic chemotherapies are often ineffective for such patients, and there is an urgent need for personalized medicine. Unlike other types of cancer, personalized treatments for pancreatic cancer are still in development. Consequently, pancreatic cancer is less sensitive to anticancer drugs and is often refractory to common treatments. Therefore, advances in personalized medicine for pancreatic cancer are necessary. This review examined advances in personalized medicine for pancreatic cancer, including the use of endoscopic ultrasound (EUS)-guided sampling. EUS-guided sampling is widely used for diagnosing pancreatic tumors and is expected to be applied to sampled tissues. Additionally, there has been an increase in clinical research using EUS-guided sampling. The combination of precision medicine using genomic testing and pharmacological profiles based on high-throughput drug sensitivity testing using patient-derived organoids is expected to revolutionize pancreatic cancer treatment.

## 1. Introduction

The number of patients diagnosed with pancreatic duct tumors is increasing annually. Currently, pancreatic ductal adenocarcinoma (PDAC) is the seventh leading cause of cancer death worldwide, with an estimated 432,000 new cases and 459,000 deaths yearly [1]. Pancreatic malignancies are difficult to detect at an early stage; patients are often diagnosed in advanced stages, and many patients receive chemotherapy [2]. However, patients with pancreatic malignancy have a five-year survival rate of less than 10%, even when treated [3]. These tumors have been classified as the most intractable malignant tumors with low antineoplastic sensitivity, and the development of innovative diagnostic techniques and treatments is required.

Since chemotherapy is administered uniformly based on the results of clinical trials, therapeutic effects vary from patient to patient. This may be because the efficacies of anticancer drugs differ due to genomic differences, even in patients with the same cancer [4]. Therefore, there is a limit on improving clinical results of anticancer drugs for advanced PDAC, and there is a need for personalized treatments for patients with PDAC.

Endoscopic ultrasound (EUS)-guided sampling, which can reliably collect tissue from malignant tumors of the bile duct and pancreas before administering chemotherapy, has been developed to facilitate the tissue collection of malignant tumors since the 1990s [5]. With the development of needles and the availability of EUS worldwide, EUS-guided sampling has been used in actual clinical settings. EUS-guided sampling has the potential to advance personalized treatment by allowing the assessment of genomic alterations and drug sensitivity [6,7,8].

Neoadjuvant chemotherapy has been common for operable or borderline PDAC [9]. In the future, the need for EUS-guided sampling will increase as the number of upfront resections for early PDACs without pathological confirmation decreases. EUS-guided sampling allows the collection of tissue aspirations by inserting a thin needle into the lesion using real-time endoscopic ultrasonography. Initially, various immunohistochemical biomarkers and RAS gene mutations were used as diagnostic aids. However, in addition to classical tissue hematoxylin-eosin staining and Papanicolaou staining, other diagnostic methods using EUS-guided samples have made remarkable progress [8,10,11]. The utility of sensitivity testing using 2D-cultured cancer cells of individual patients with PDAC has been examined, however this method was found to have poor clinical utility due to poor establishment efficiency [12].

Next-generation sequencing (NGS) that collectively captures cellular activities using conventional methods, such as polymerase chain reaction, Western blotting, and enzyme-linked immunosorbent assays that examine a single gene or protein have been proposed for analyzing tumor tissue [13]. The invention of organoid technology, which enables cancer and normal cell culture biopsies with high efficiency and greatly influences modern biological research, has led to great advances in medical technology. High-throughput methods have also been developed for drug sensitivity testing, and technological innovations are occurring rapidly. By applying these technologies, various studies using cancer tissues are currently being actively conducted [14,15,16].

Here, we summarize recent studies on personalized medicine for PDAC and show recent findings regarding the application of EUS-guided sampling to personalized medicine.

## 2. Personalized Cancer Treatment Using Genomic Profiling

### 2.1. The Current Status of Cancer Precision Medicine

Traditional anticancer drugs act on all rapidly dividing normal cells and cancer cells. Therefore, the approach to treatment is shifting to genetically modified strategies that identify potential therapeutic targets with enhanced specificity. However, it is necessary to conduct genotype-matched clinical trials, such as basket and umbrella studies, to confirm the usefulness of cancer precision medicine [17,18]. Several large basket trials aimed at studying different cancer types, such as the Targeted Agent and Profiling Registry, are currently underway and are expected to provide genotype-matched treatment strategies [19,20,21,22,23]. 

Genomic sequencing assays for tumor analysis are a recent development in the field of cancer treatment [13,24]. The increased worldwide availability of NGS in recent years has facilitated the analysis of genomic biomarkers. Precision medicine based on genomic biomarkers is perhaps most frequently used to treat lung adenocarcinoma [25]. Genomic tests for EGFR mutations [26], ALK fusions [27], ROS1 fusions [28], and BRAF mutations are widely used as clinical tests, and corresponding molecular-targeted therapies have become the standard treatment for patients with lung cancer. Moreover, high microsatellite instability and mismatch repair deficiency have been utilized as biomarkers for programmed cell death 1 inhibitors, and NTRK fusions have been utilized as tumor-agnostic genomic biomarkers for TRK inhibitors [29]. 

In a recent large cohort study of 10,000 cancer patients using the MSK-IMPACT platform, 37% of patients had actionable genetic changes. However, since then, only 11% have been enrolled in genotype-matched clinical trials [30]. Conversely, treatment with molecularly matched therapies has been reported to significantly improve survival outcomes in patients with pancreatic cancer, based on the ongoing Know Your Tumor program, including 1000 patients with PDAC [4].

### 2.2. Using EUS-Guided Sampling to Improve Personalized Treatment for PDAC 

There are some unresolved issues regarding genomic testing for PDAC in clinical practice. It is often possible to obtain sufficient surgical specimens and biopsy samples for other tumors, however the difficulty in obtaining pancreatic tumor samples is a major problem. EUS-guided sampling has been used to collect tumor samples from the pancreas since the 1990s and has become widely used as the first choice for sampling PDACs [31]. 

EUS-guided fine-needle aspiration (FNA) was first described in 1993 by Vilmann et al. as a technique to collect samples for smear cytology [32]. Later, this technique was also used to obtain material for histological and immunohistochemical samples, as well as for molecular processing (cell block technique, tissue core acquisition). In some studies, >80% of EUS-FNA samples were adequate for histology and immunohistochemistry [33,34,35]. In the early years of this century, a new approach to EUS-guided sampling was initiated: to yield intact tissue cores with specifically designed needles called EUS fine-needle biopsy (FNB). The first generation of these core needles was based on the trucut biopsy technique (QuickCore, Wilson-Cook, Winstom Salem, NC, USA) [36], but results were limited by technical problems. Second generation needles with side-hole bevels (ProCore, Wilson-Cook) [37] and third generation needles with Franseen-type bevels (Acquire, Boston Scientific, Marlborough, MA, USA) [38] or fork-tip type bevels (SharkCore, Medtronic, Minneapolis, MN, USA) [39] proved to be more effective in terms of larger amounts of cohesive tissue with preserved tissue architecture and fewer needle passes needed to obtain samples adequate for diagnosis. Furthermore, the large amount of core tissue obtained from PDACs provides an opportunity for immunostaining, histologic diagnosis, and molecular and genetic analyses for personalized treatment.

Currently available cancer genomic profiling tests, such as the FoundationOne CDx (Foundation Medicine, Cambridge, MA, USA), which is a qualitative NGS-based in vitro diagnostic test that uses high-throughput hybridization-based capture technology to detect large amounts of genomic information, usually requiring 10–500 ng of DNA. However, it is still unclear whether EUS-guided sampling is the optimal method for collecting tumor samples for genomic sequencing of PDACs [10,11,40]. For genomic analysis, the collected sample is fixed with formalin and laser capture microdissection is performed to extract DNA. Archival samples, like formalin-fixed paraffin-embedded tissue obtained from EUS-guided sampling, are often unusable due to problems such as low quantity or quality of tissue and reduced-quality DNA [7,11]. It is necessary to identify a method of collecting samples for genomic testing and diagnosis rather than using archived samples.

Successful molecular biological analysis from fixed formalin specimens of cell specimens collected by EUS-FNA was reported in 2010 by Fujita et al. [41]. In 2012, Bournet et al. extracted RNA directly from samples collected by EUS-FNA and EUS-FNB for molecular subtyping of PDACs [11]. In addition, the first attempt to evaluate the chemotherapy-sensitivity to PDACs using RNA extracted from EUS-FNA was reported in 2010 by a Japanese group [42]. It has been reported that greater DNA yield can be collected by freezing the collected sample and extracting the DNA [6]; this is expected to be applied in the future. Due to low DNA quality, additional tissue sampling is often required in the clinic. Published studies with two novel needles, Franseen and fork-tip needles, demonstrated a significant difference in terms of sample adequacy, accuracy, or acquisition of histologic materials when compatible with standard EUS-guided sampling [43]. Additionally, a randomized study demonstrated higher histologic yield, tumor cellularity, and surface using EUS-FNB needles [44]. Several studies have reported a higher success rate of NGS using EUS-guided samples (Table 1). Larson et al. reported an NGS success rate of 70.4% using EUS-FNB, compared to only 42.9% when using EUS-FNA. Elhanafi et al. reported that the success rate of NGS using FNB samples was 90.9%, much higher than the 66.9% that was achieved when using FNA [10]. Therefore, we recommend the FNB needle to collect DNA for genomic testing. On the other hand, some studies, including pharmacological profiling, have shown that “real-world” EUS-FNA samples can be used for genome profiling using NGS [6,8]. A recent study showed no superiority of EUS-FNB over EUS-FNA for RNA extraction from PDACs [45]. Therefore, further research is needed to determine which sampling (type of puncture needle, gauge, amount of tissue to be collected, suction method, etc.) and specimen processing methods are suitable for obtaining good-quality RNA and DNA samples [40]. 

### 2.3. Cancer Genomic Medicine for PDAC: A Future Perspective

PDAC has been reported to have only a few druggable gene alterations. Two large cohort studies have investigated the status of PDAC mutations using genomic analysis. Although *KRAS*, *CDKN2A*, *TP53*, and *SMAD4* have been identified as the major driver genes for PDAC, detection rates for other genetic alterations have been reported to be very low [48,49,50]. A retrospective study examined 336 PDAC patients who underwent genomic sequencing to identify gene mutations. Only 1% of these patients reported receiving consistent treatment based on sequencing results. From these results, the authors concluded that the practical application of precision medicine for PDAC is currently limited [51].

Precision medicine for PDAC is currently still under development. For example, targeting genetic alterations associated with homologous recombination deficiencies such as *BRCA1/2, PALB2, FANC*, and *ATM* has been reported to be a promising therapeutic strategy for PDACs that are sensitive to poly (ADP-ribose) polymerase (PARP) inhibitors and platinum [49]. The PARP inhibitor Olaparib was recently approved to be used in maintenance therapy after platinum-based treatment in PDAC cases with germline BRCA mutations [52]. The further development of a comprehensive gene panel containing genes specific to PDAC and associated with predictive biomarkers for DNA damage repair and immunotherapy is expected.

## 3. Personalized Cancer Treatment Using Pharmacological Profiling

### 3.1. Effectiveness and Role of Chemosensitivity Tests for PDAC Treatment

Although gene mutations promote tumor progression, some reports have suggested that they may be less associated with chemotherapy resistance [53,54,55]. One study found that global genomic features cannot classify tumors or predict therapeutic responses [53]. Similar results were found in another report of a larger cohort of patients using the whole-genome sequencing approach [54]. Sensitivity studies to numerous drugs have been reported in 28 PDAC patient-derived cell lines and xenografts; however, there was a correlation between therapeutic efficacy and gene mutation in only one case [55]. These data highlight the risk of relying on genetic analyses alone to predict the efficacy of currently available drugs for PDAC. Alternatively, sensitivity profiling that assesses cell viability by administering therapeutic agents to the patient’s cancer-derived tissue has been suggested to provide useful information for personalized PDAC treatment. However, attempts to assess drug sensitivity using patient-derived “cell lines”, have not been successful.

Precision medicine approaches for PDAC are challenging because of the short median survival of patients with metastatic PDAC. Furthermore, sensitivity testing is difficult for the following reasons: (1) difficulty in establishing cell culture [12]; (2) the long time required to get results [56]; (3) sequential tumor chemosensitivity changes due to treatment with chemotherapy [57]; and (4) individual tumor heterogeneity [58]. To effectively assess chemosensitivity for PDAC, it is necessary to have good access to repeated tissue samplings at multiple sites, depending on the patient’s clinical course, and obtain a high culture success rate and more rapid test results. Therefore, technology that overcomes these difficulties is necessary.

### 3.2. Patient-Derived Tumor Organoids for PDAC: A Future Perspective

To evaluate the drug sensitivity of pancreatic malignancies, it is necessary to establish a more efficient method for culturing cancer cells. The cancer stem cell hypothesis states that there is a small subpopulation of cells in tumors with a capability of self-renewal and tumorigenicity. However, attempts to culture those cancer stem cells have been unsuccessful. In 2007, it was reported that colon cancer stem cells could be cultured using the sphere culture method [59], but the success rate was low (20–30%), which is not practical for clinical use. The organoid culture system, developed by Sato et al. in 2009, made it possible to expand normal and cancer adult cells. Organoids are thought to be self-organized from single stem cells, which phenotypically and molecularly mimic the origin of tissues and organs. This technology is a powerful tool because it can be applied NGS and genomic modification using CRISPR-Cas9 technology. The applications of organoids have advanced to evaluate gastrointestinal membrane permeability in two-dimensional cultures [60]. Additionally, the success rate of PDAC organoid establishment is much higher than that of primary cell cultures [61]. Furthermore, high-throughput methods to examine drug sensitivity in organoids have been developed, requiring only six weeks to complete [62]. If an effective drug can be identified more quickly, it may be possible to select the appropriate treatment for initial chemotherapy.

Patient-derived organoids (PDOs) have the potential to be used as important tools for personalized cancer treatment [63]. Basic cancer research using PDOs was first reported in our institution [61,64,65]. Many studies have reported that PDOs are good models for drug screening and predicting the response to treatments [14,62,66]. Additionally, pharmaceutical profiling through EUS-guided sampling and PDOs is likely to be useful for improving PDAC treatment, targeting vulnerabilities of tumors in individual patients. EUS-guided sampling can be repeatedly performed with low risk of complications, making it possible to obtain samples from liver metastases and ascites as well as the primary lesion [66].

Whether the clinical implementation of PDOs for drug sensitivity tests is useful has not been fully evaluated for PDAC. However, irradiation/drug tests using rectal cancer PDOs were reported to have an accuracy of 84.4% in the clinical treatment course. In most rectal cancer cases, PDO response analysis was completed in less than four weeks, enabling treatment recommendations to be generated within a clinically meaningful time frame [67]. If these results can be applied to PDAC cases, they are expected to be very useful for personalized medicine.

### 3.3. EUS-Guided Sampling for Personalized Medicine Using PDOs

Only ~20% of patients with PDAC are eligible for surgical intervention [68], and sufficient tissue could be available, making it easy to diagnose and culture PDOs. By applying EUS-guided sampling to inoperable patients, it has become possible to overcome the limitations of taking samples from PDAC tumors and obtain the necessary tissue. PDOs can be cultured by collecting a small amount of tissue separately from the pathological diagnostic sample, which does not interfere with clinical examination in the actual clinical setting. EUS-guided sampling can also be safely performed in patients at any stage of PDAC as well as preoperatively, before and after radiation and chemotherapy, and after recurrence.

Recently, there have been increasing numbers of reports on how to effectively collect specimens to establish organoids (Table 2). FNB-based tissue sampling has become more common than FNA-based cytology for diagnosing PDAC. Many studies have suggested the usefulness of 22-gauge FNB needles for PDAC diagnosis [69] because this size of FNB needle allows for core tissue samples with fewer passes. Aspiration of core tissue with preserved architecture is also beneficial for diagnosis and obtaining a sufficient amount of sample for the organoid establishment and molecular genetic studies. The success rate of PDO establishment from tissues obtained through EUS-guided sampling is reported to be about 60% to 82% [61,62,70,71,72,73,74], which is the same success rate as with surgically resected tissues. Furthermore, there is no significant difference in the establishment efficiency between single-pass and double-pass punctures, therefore single-pass punctures with a 22-gauge FNB needle are recommended [74]. However, there are still only a few reports regarding the method of EUS-guided sampling for PDO establishment; therefore, further research is needed.

### 3.4. Human PDOs Establishment from EUS-Guided Sampling

Methods for establishing PDAC organoids have been reported by multiple research groups. In the field of PDAC-related organoid research, the first organoid was generated from mouse pancreatic tissues [63,64]. Following this, the creation of organoids derived from human PDAC has been achieved via various methods. Some groups embed cells in Matrigel overlaid with culture medium, while others culture cells on a Matrigel bed covered with a mixture of Matrigel and culture medium. Matrigel serves as the scaffold for epithelial cells because it includes multiple extracellular matrix proteins, some of which are members of the basal membrane.

There have also been several reports on how to generate PDOs from PDAC specimens collected by EUS-guided sampling. The Tuveson research group [62,63], which has created the largest PDAC organoid library, has reported a detailed method for PDO establishment from the small amount of needle biopsy samples. Tiriac et al. described the method as follows [8]. Tissue samples collected by EUS-guided sampling were usually contaminated with numerous red blood cells due to sample aspiration during the procedure; therefore, the specimens needed to be treated with blood cell lysis to remove them and then mechanically dissociated in digestion media. These tissue fragments were then plated with dome-type Matrigel and overlaid with a culture medium. The addition of niche factors (EGF, FGF10, BMP inhibitor, TGF-beta receptor inhibitor, Wnt3A, and Rspondin1) to the culture medium to maintain the stemness of the PDOs was an essential process for organoid culture. Seino et al. described that the obtained specimens were chemically dissociated into single cells before embedment in Matrigel [56]. Subsequently, the Matrigel dome was overlaid with a basal culture medium, supplemented with niche factors required for each PDO growth (Table 3).

The niche factors, which are essential for the growth and survival of PDOs are determined by each organoid culture. Normal human pancreas-derived organoids require EGF, BMP inhibitor, TGF-beta receptor inhibitor, Wnt3A, and Rspondin1, while some PDAC organoids can still propagate without some of these factors due to genetic aberrations. Despite the lack of genetic abnormalities in canonical Wnt signaling pathways, comportment of the PDAC organoids could still expand in Wnt or R-spondin free environments. Seino et al. proposed the classification of PDACs based on Wnt signaling dependency and revealed that PDACs could potentially become more malignant, with independence from the stem cell niche. Our understanding of the minimum culture condition in which some of the niche factors are depleted enables us to predict the existence of genetic changes and the malignant status of original tumors.

### 3.5. High Throughput Drug Screening Test Using PDOs

First, we will discuss the high-throughput method, which has rapidly promoted the practical application of actual drug screening testing. High-throughput screening (HTS) is now being used for basic and applied research in academia [15]. It comprises the screening of large chemical libraries for activity against biological targets via the use of miniaturized assays and large-scale data analysis. A high-throughput analysis is performed using 96-, 384-, or 1536-well microtiter plates. Generally, organoid cells are embedded in a Matrigel-coated plate and cultured using a culture medium containing niche factors. After administering a serially diluted solution of each drug to the well, drug screening is performed by assaying the cell death of organoids. The luminescence ATP-based assay (CellTiter-Glo^®^ 3D cell viability assay, Promega) is reported to be used to determine the number of viable cells in 3D cell culture based on quantitation of the ATP present, a marker for the presence of active cells. Furthermore, an ATP-independent assay method to measure the activity of dead-cell protease, which is released from cells that have lost membrane integrity (CytoTox-Glo™ Cytotoxicity and CyQUANT™ assays, Promega), was also used in several reports. The CytoTox-Glo™ assay relies on the properties of recombinant luciferase, which uses aminoluciferin as a substrate to generate a stable luminescent signal.

In principle, organoids are suitable for HTS, but the technical constraints and extensive manipulation required by current methods have hampered progress toward simple clinical applications. Therefore, various simple methods have been developed, and methods for reducing costs have been reported. Fluorometric methods to quantitatively assess cell death using propidium iodide and Hoechst fluorescence in 3D in a plate reader have been reported to have significantly improved analysis time, as well as ease of use, compared to other established methods [76]. Another cell death assay method, the bright-field method, uses a simplified geometry by seeding cells around the rim of the wells (mini-rings) [15]. This method has shown that organoids can be assayed on the same seeded plate, require fewer cells, and can be easily automated for HTS.

The proper timing for assaying cell death after drug administration remains unknown. Most reports have examined cell death approximately 1–5 days after drug administration. There has been not standardized method for HTS using PDOs, but it is expected that a consensus will soon be reached. There have been few actual reports on PDAC cases (Table 4), and further technological innovation is required for practical use.

### 3.6. Drug Screening Test for PDAC Using Organoids

The feasibility of a drug screening study using PDOs was first shown in metastatic, heavily pretreated colorectal and gastroesophageal cancer patients recruited in phase I/II clinical trials. They showed PDOS drug screening with 100% sensitivity, 93% specificity, 88% positive predictive value, and 100% negative predictive value in forecasting response to targeted agents or chemotherapy in patients [16]. Several pilot studies on PDAC drug screening related to clinical data have been vigorously pursued based on this milestone study [56,62,77]. The report by Tiriac et al. is currently the largest study of drug screening using PDOs [62]. They performed drug screening of five anticancer drugs for 63 cases of PDOs. The sensitivity to each drug was evaluated in three divisions, and the efficacy in the clinic was shown, although it included a small number of cases. They combined drug screening data from PDOs with genetic data to create original indexes. These indexes indicated that tumor responsiveness to FORFIRINOX was superior in the oxaliplatin-sensitive group, but there was no difference in susceptibility to 5-FU and SN-38 (irinotecan). The study also presented the course of treatment in one case of PDAC. The results of the drug screening study using organoids established multiple times in chronological order from this case reflected the tumor profile in actual clinical practice. It demonstrated the usefulness of time-series organoid drug screening. Moreover, Driehuis et al. showed the usefulness of the drug screening test in a limited number of four patients who created PDOs [56]. These two studies involving numerous PDO cell lines and patient follow-ups demonstrated a significant correlation between the PDAC PDO response and the clinical patient’s response for the gemcitabine–paclitaxel and FOLFIRINOX treatments, despite the small sample size.

Wolff et al. followed one patient with PDAC for five years, with clinical records, pathology, dynamic evolution of molecular and cellular changes, chemotherapy and targeted therapy with PDOs culture over time, and the response to treatment with immunotherapy [57]. Although this only followed one patient, detailed tumor profiles over time demonstrated the usefulness of drug screening with PDOs.

In addition, Frappart et al. also reported a drug screening study for 22 anticancer drugs in patients with PDAC [77]. In the representative cases presented in this study, the screening test result was clarified five days after the initial treatment of FORFIRINOX for metastatic PDAC. Drug screening testing revealed that paclitaxel and gemcitabine were significantly more effective than other therapeutic agents. The tumor diameter increased by 29% after the initial FORFIRINOX treatment, but there was a significant therapeutic effect by changing to gemcitabine + paclitaxel treatment in this case.

As described above, PDAC drug screening has not yet been totally successful. This may be related to the lack of effective anticancer drugs and the microenvironmental factors of tumors exhibiting strong fibrosis. In addition, as described in the previous section, a method for determining the therapeutic effect of high-throughput drug screening for PDAC organoids has not been implemented in clinical practice. To implement PDOs drug screening in actual clinical practice, a large number of studies and development of more effective anticancer agents are required in metastatic PDAC cases.

## 4. Additional Diagnostic Methods for PDACs

The amount and quality of tissues extracted using EUS-guided sampling are frequently insufficient for histopathological diagnosis, despite improved puncture techniques and needles. New diagnostic methods in addition to EUS-guided sampling, such as microRNA-based tests [78], confocal laser endomicroscopy [79], and proteomics-based tests [80], have been reported to have potential for future clinical application. A recent development in this field is the use of lipid profiling by probe electrospray ionization mass spectrometry and machine learning, including artificial intelligence for diagnosis. Specifically, probe electrospray ionization mass spectrometry (PESI-MS) can rapidly achieve direct mass spectrometry using a very fine needle to collect a small amount of tissue without pretreatment. It also detects low molecular weight metabolites and lipids, which provide important information for disease detection. The accumulation of spectrum patterns for machine learning may allow rapid diagnosis of tumors or accurate identification of the sample within several minutes. Furthermore, PESI-MS has been reported to be effective for the diagnosis of head and neck squamous cell carcinoma [81], hepatocellular carcinoma [82], breast cancer, and renal cell carcinoma [83]. In several studies with other tumors, PESI-MS can be used for rapid diagnosis in minutes. Using EUS-guided sampling of PDAC for PESI-MS can be a faster diagnostic method than pathological diagnosis which can take days. Additionally, rapid blood-based diagnostic modalities to detect PDAC with high accuracy have been recently reported [84]. By applying more cases to machine learning and utilizing artificial intelligence, there is a possibility that subtypes of PDAC can be detected and used to improve personalized medicine in the future.

## 5. Conclusions

Personalized medicine for PDAC is still in development. Consequently, PDAC has a low sensitivity to anticancer drugs and is typically intractable. However, advances in personalized cancer care for PDAC will allow improved chemotherapy selection and more effective patient selection, as well as facilitate the identification of patients in whom chemotherapy may be ineffective, thus avoiding unnecessary treatment. It is expected that the combined use of gene profiling based on genetic testing and pharmaceutical profiling using HTS and PDOs will revolutionize PDAC treatment.

We recommend the use of FNB needles to collect DNA for genomic testing because of its ability to extract more tissue. Clinical studies on the diameter of needles, number of needle passes, and processing techniques are needed. Additionally, EUS-guided sampling is expected to help identify changes in tumor sensitivity over time and identify patient-specific response to chemotherapy. EUS-guided sampling has few complications and allows for repeat tissue sampling. Therefore, it is expected that regular PDO preparation using EUS-guided sampling during the course of treatment will improve treatment accuracy.

## Figures and Tables

**Table 1:** Precision medicine for pancreatic ductal adenocarcinoma (PDAC) using endoscopic ultrasound (EUS)-guided sampling.

Authors and Year	Method of Sampling and Type of Needle	Number of Patients	Successfully Sequenced Samples, n (%)
Larson et al. [10], 2018	EUS-FNB EUS-FNA Percutaneous	54 7 8	38 (70.4%) 3 (42.9%) 8 (100%)
Hayashi et al. [40], 2018	EUS-FNA (FFPE) 22-G	9	7 (78%) Re-biopsy 2 (22%)
Elhanafi et al. [46], 2020	EUS-FNB 22-G EUS-FNA 22-G	22 145	20 (90.9%) 97 (66.9%)
Semaan et al. [47], 2020	EUS-FNA (cytology)	ND	23 (ND)
Kandel et al. [6], 2020	EUS-FNB (fresh frozen) EUS-FNA (fresh frozen) 19-G for body and tail/22-G for head	50 50	39 (78%) 7 (14%)

EUS-FNA: endoscopic ultrasound-guided fine-needle aspiration, EUS-FNB: endoscopic ultrasound-guided fine-needle biopsy, FFPE: formalin-fixed paraffin-embedded, ND: not determined: PDAC, pancreatic ductal adenocarcinoma.

**Table 2:** Culture of patient-derived organoids.

Authors, Year	Method of Sampling/Type of Needle	Number of Patients	Number of Organoids Created n (%)
Boj et al. [70], 2015	FNA/ND	ND	2 (primary and metastasis)
Tiriac et al. [62], 2018	FNB/22-G	60	43 (71%)
Seino et al. [61], 2019	FNB/22-G	ND	27 (ND)
Bian et al. [71], 2019	FNB/ND	ND	24 (85%)
Henning et al. [75] 2019	FNA/ND	6	5 (83%)
Vilgelm et al. [72], 2020	FNA/25-G	5	5 (ex vivo tumor, 100%)
Juiz et al. [73], 2020	ND	ND	20 (ND)
Lacomb et al. [74], 2020	FNB/22-G 1 pass FNB/22-G 2 pass	25 42	22 (88%) 34 (81%)

EUS-FNA: endoscopic ultrasound-guided fine-needle aspiration, EUS-FNB: endoscopic ultrasound-guided fine-needle biopsy, ND: not determined.

**Table 3:** Methods to culture organoids from human PDAC using EUS-guided sampling.

Authors, Year	ECM-Matrix	Medium
Tiriac et al., 2018 [8]	Matrigel 100% Dome-type	Advanced DMEM/F12, HEPES (10 mM), Glutamax (1X), A83-01 (500 nM), hEGF (50 ng/mL), mNoggin (100 ng/mL), hFGF10 (100 ng/mL), hGastrin I (10 nM), N-acetylcysteine (1.25 mM), Nicotinamide (10 mM), PGE2 (1 μM), B27 supplement (1X), R-spondin-1 (10%), Afamin/Wnt3A (50%).
Seino et al., 2018 [61]	GFR-Matrigel 100% Dome-type	Advanced DMEM/F12, HEPES (10 mM), Glutamax (2 mM), B27 (1X), Gastrin I (10 nM), N-acetylcysteine (1 mM), mEGF (50 ng/mL), mNoggin (100 ng/mL), R-spondin-1 (10%), Afamin-Wnt-3A (25%), A83-01 (500 nM), SB202190 (10 μM).
Bian et al., 2019 [71]/Juiz et al., 2020 [73]	GFR-Matrigel 100% Dome-type	Advanced DMEM/F12, HEPES (10 mM), Glutamax (1X), hFGF10 (100 ng/mL); hEGF (50 ng/mL), hNoggin (100 ng/mL), Wnt3a (30%), R-spondin-1 (10%), hGastrin I (10 nM), Nicotinamide (10 mM), N-acetylcysteine (1.25 mM), B27 (1X); A83-01 (500 nM); Y27632 (10.5 µM).
Hennig et al., 2019 [75]	GFR-Matrigel Dome-type	DMEM/F12, Wnt3a (50%), HEPES (1X), Pen/Strep (1X), and 1x Glutamax (1X), Noggin (10%), R-spondin-1 (10%), B27 (1X), Nicotinamide (10 mM), gastrin (1 nM), N-acetyl-L-cysteine (1 mM), Primocin (1 mg/mL), mEGF (50 ng/mL), hFGF10 (100 ng/mL), A-83-01 (0.5 μM), N2 (1X).

DMEM: Dulbecco’s modified Eagle’s medium; EGF, epidermal growth factor; FGF, fetal growth factor; GFR, growth factor reduced; PDAC, pancreatic ductal adenocarcinoma.

**Table 4:** Methods of high-throughput drug screens for patient-derived organoids (PDOs) of PDAC.

Authors, Year	Plate	Number of Seeded Cells	Timing of Assay	Assay Method	Target Agents	No of PDOs
Tiriac et al., 2018 [8]	96 well	500 cells/well	5 days after administration.	CellTiter-Glo^®^ (luminescence ATP)	5	66
Driehuis et al., 2019 [56]	384 well	ND	3 days after administration.	CellTiter-Glo^®^ (luminescence ATP)	76	24
Frappart et al., 2020 [77]	96 well	2000 cells/well	4 days after administration.	CytoTox-Glo^TM^ (luminescence non-ATP)	22	21

ND, not determined; PDAC, pancreatic ductal adenocarcinoma; PDO, patient-derived organoids.
